# First reported case of Lyme carditis in Southwest Michigan

**DOI:** 10.51894/001c.5933

**Published:** 2017-02-02

**Authors:** Nicholas M. Frazier, Richard W. Douce

**Affiliations:** 1 Internal Medicine Resident, Lakeland Regional Medical Center, Saint Joseph, MI; 2 Core Faculty and Attending Physician Infectious Diseases/Internal Medicine, Lakeland Regional Medical Center, Saint Joseph, MI

**Keywords:** tick-borne pathogens, heart block, lyme disease, lyme carditis

## Abstract

Lyme disease is the most common tick-borne infection found in the eastern United States. In recent years, it has become an emergent Michigan public health concern. Lyme carditis is a recognized rare complication which is classically characterized by rapidly fluctuating degrees of heart block. In severe cases, or if inappropriately treated, Lyme carditis can also result in profound bradycardia, perimyocarditis, and sudden cardiac death. This report describes the first documented case of third degree heart block associated with Lyme carditis to occur in Michigan. This is a retrospective case report of a patient evaluated and treated for Lyme carditis in Southwest Michigan in July, 2016. All information was obtained from either the patient or his electronic medical record. Despite initial misdiagnosis and inappropriate management, this patient ultimately received more appropriate medical therapy within 24 hours of first presentation. After eight days of high dose intravenous Ceftriaxone and supportive care, and more than two weeks of oral Doxycycline, the patient’s symptoms resolved and the disease was treated to resolution. Neither permanent nor temporary pacing was needed during/after the course of treatment. When correctly identified, Lyme disease and Lyme carditis can be easily treated. Although this patient’s history was without reported tick bite or exposure to a known host for Lyme disease, the authors believe that the patient’s history and physical exam was definitive enough to warrant the start of IV therapy with telemetry monitoring upon first presentation. The fact that the condition was not first diagnosed by providers indicates a potential gap in medical knowledge and awareness that should be addressed in clinical practice. The authors consider this case a harbinger of the emerging disease of Lyme carditis. Physical exam and EKG findings should guide clinicians’ therapeutic approaches. Although treatment with appropriate antibiotics is typically curative, therapeutic delays can lead to deadly results.

## INTRODUCTION

Lyme carditis is a rare complication of Lyme disease occurring in about 1% of cases.^1^ The complication typically develops within the first two months of infection, and is characterized by rapidly fluctuating classes of atrioventricular (AV) block.^2^ Typical symptoms including erythema migrans (i.e., rash), fatigue, fever, myalgias (i.e., muscle pain) and arthralgias (i.e., joint pain), and are often present in some combination.

Despite purported underreporting of Lyme disease,^3,4^ there were over 251,000 confirmed cases reported to the Centers for Disease Control and Prevention from 2005-2014,^5^ making it the most common tick-borne disease in the United States. During this period, 96% of cases were confined to 14 states. The State of Michigan accounted for only 0.32% of all confirmed cases.

**Figure 1:**
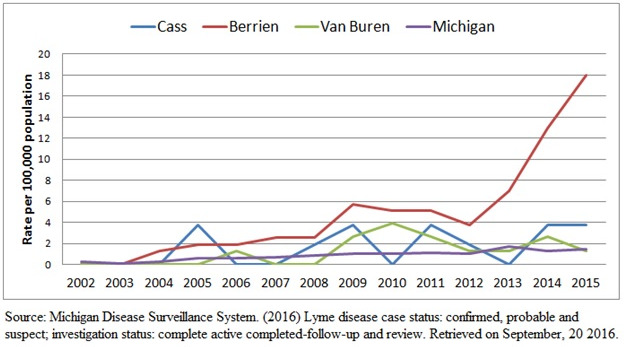
Michigan Lyme Disease Incidence Rates (2002 – 2015).

In recent years, there has been an increased incidence of Lyme disease in Michigan and nationally^6^ (see Figure 1), making this tick-borne pathogen an emergent public health concern. This increase has been confirmed in the western and northern portions of Michigan, where *Borrelia burgdorferi* is endemic.^7,8^ The etiology for this trend are multifactorial. However, the northward expansion of the primary vector (or organisms responsible for transmission) for Lyme disease, the tick *Ixodes scapularis*,^9^ as well as the possible emergence of a new tick species carrying Lyme, are also possible contributors. Despite this increased incidence, the authors believe this is the first published account of Lyme carditis in Michigan resulting in complete heart block.

### Case Presentation

This patient was a 36-year-old Caucasian male who first presented to an emergency department (ED) at a small community hospital a week after visiting Michigan's upper peninsula. His chief complaint of three days was of low back and bilateral leg pain associated with intermittent night sweats and fever. Although the patient had been working outside as a concrete pourer, he had not spent time in the woods nor near wildlife. The patient’s past medical history was significant for dyslipidemia and tobacco use. His father and brother had significant cardiac disease, with his brother having sustained a myocardial infarction (heart attack) when just 28 years old.

**Figure 2:**
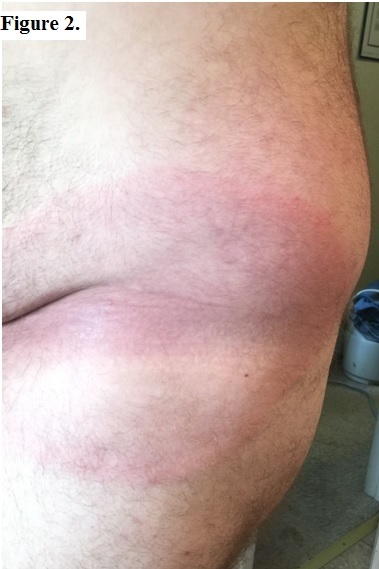
Anterior left hip and groin, 26cm. erythematous rash with central clearing.

Per the original ED records, initial physical examination was only pertinent for mildly diaphoretic skin with an egg-shaped erythematous rash on his anterior left hip and groin measuring 26 cm. with central clearing, or area void of red coloration (see Figure 2). The rash was neither tender nor warm to palpation. The patient had intermittent bradycardia in the 50s, and his vital signs were otherwise within normal limits.

Due to the patient’s reported chest discomfort, an EKG (electrocardiogram) was completing, showing 2^nd^ degree AV block (Mobitz I) with heart rate of 64. Three serial troponin labs were drawn to evaluate for possible heart muscle damage but found to be negative. A nuclear exercise stress test was then done but was non-diagnostic due to the patient not reaching target heart rate. The inflammatory markers Erythrocyte Sedimentation Rate (ESR) and C-Reactive Protein (CRP) were noted to be elevated, suggesting an inflammatory and possibly acute infective process. Therefore, a Lyme serology was ordered. The patient was prescribed oral Amoxicillin for possible Lyme disease and discharged home.

Within a few hours after leaving the ED, the patient developed substernal chest pains, nausea and vomiting and sought re-evaluation at Lakeland Regional Medical Center in St. Joseph, MI. Initial examination revealed an anxious but fit-looking man in mild distress. The patient’s exam demonstrated bradycardia with pulse in the 50s, a regular rhythm and without murmur. The aforementioned rash was present and unchanged.

A repeat EKG now showed 3rd degree heart block with new T-wave inversion in leads II, III, and aVF, suggestive of possible inferior ischemia. The patient reported improvement in his chest pain while in the ED, but had mildly elevated troponins of 0.05 ng/mL at three separate times, suggestive of some level of cardiac damage. ESR and CRP levels were elevated at 81mm/h and 7.1mg/dL respectively, again signifying an acute inflammatory process. The Cardiology service was consulted and therapy was initiated for possible acute coronary syndrome (ACS), with the patient receiving full dose Aspirin and IV heparin. The patient was ultimately admitted to the cardiac/telemetry floor for 3^rd^ degree heart block, bradycardia and chest pain.

Overnight, the patient experienced significant subjective improvements of his myalgias and arthralgias without fever. Although the patient’s rash was somewhat improved, a new systolic murmur was appreciated on cardiac exam. Telemetry demonstrated fluctuations between 1^st^ and 3^rd^ degree AV block, with 3rd degree being dominant.

Due to the fluctuating heart block and the patient’s presenting symptoms, an intravenous (IV) Ceftriaxone 2g daily dose was started by the hospitalist team to treat presumptively for Lyme disease as clinicians awaited serology results. In view of the patient’s family history, labs values and EKG abnormalities, the Cardiology service felt that ACS could not be ruled out. An echocardiogram showed a normal left ventricular ejection fraction (i.e., percentage of blood leaving the heart each time it contracts) of 60-65% and moderate left atrial enlargement. A cardiac catheterization was scheduled and temporary pacing was discussed and made available if needed.

The cardiac catheterization was performed and demonstrated only mild coronary artery disease which required no further intervention. Shortly after catheterization, Western Blot testing from the patient’s original ED visit resulted demonstrating positive IgM (p41 and p23) and IgG (p18, p23, p39, p41, p45, and p66) bands. In light of these findings and a largely negative cardiac work-up, a diagnosis of Lyme carditis was made. In house serologic testing confirmed this diagnosis one day later.

Due to the patient’s worsening cardiac function with plans to transition to oral antibiotics on discharge, an additional Doxycycline 100mg every 12 hours order was added to the existing Ceftriaxone regimen. All eventual blood cultures results were negative. During the next six days, the patient's heart rate and conduction abnormalities gradually improved.

By hospital day eight, the patient remained bradycardic in the high 50s but was now in 1^st^ degree AV block. The patient was discharged on oral Doxycycline 100mg twice daily for 14 to total 21 days of antibiotic therapy and scheduled for a cardiac follow-up appointment. A week later, the patient was asymptomatic and in normal sinus rhythm without any conduction abnormalities. The patient was instructed to finish the remaining course of oral antibiotics and make aggressive lifestyle changes to decrease his underlying cardiac risk factors.

## DISCUSSION

This patient’s presentation with erythema migrans, fever, chills, myalgias, and arthralgias^8^ as well as rapidly fluctuating AV block^2^ was classic for Lyme carditis. Although cases of severe conduction abnormalities have been documented in top endemic areas, this case represents the first published account of complete heart block related to Lyme infection occurring in Michigan. As the incidence of Lyme disease continues to rise, so should the incidence of Lyme carditis.

Although Lyme carditis can be fatal, it generally carries a good prognosis, especially when appropriate antibiotic therapy is administered early. Based on the severity of conduction abnormalities, patients should be treated with either oral or IV antibiotics for a total of 21-28 days to minimize cardiac abnormalities and prevent future complications. For patients with mild disease (1st degree AV block with PR interval <300 milliseconds), oral agents such as Doxycycline 100mg twice daily, Amoxicillin 500mg three times daily, or Cefuroxime 500mg twice daily and outpatient follow-up can be appropriate. However, adults who are symptomatic, have 2nd or 3rd degree AV blocks, or have a PR interval >300 milliseconds on EKG should be hospitalized for telemetry monitoring and administered daily IV Ceftriaxone 2 grams until conduction abnormality improves.^10^

Typically, patients’ severe AV block improves to a lesser degree block within one week.^11^ In rare cases, Lyme carditis has been implicated in nonreversible AV block^12,13^ and even more infrequently with sudden cardiac death.^14–16^ Due to the reversible and typically short-lived nature of conduction abnormalities, permanent pacing is not generally needed^17^ unless advanced heart blocks fail to improve after six weeks of therapy.^18^ If the need for temporary pacing arises as seen in up to 40% of severe cases,^2^ percutaneous or modified temporary transvenous pacing should be used.^17^

Despite this patient presenting with classic features of Lyme carditis from a diagnosis of Lyme disease, optimal therapy was not initiated at his onset of care. Instead of being admitted and started on IV antibiotics due to his 2nd degree AV block, the patient was treated as an outpatient with oral Amoxicillin and symptom relief. Whether the patient would have transitioned from 2^nd^ degree to complete heart block if he had been started on IV Ceftriaxone on first presentation is uncertain. Nevertheless, our hope is to prevent future delays in optimal management by increasing clinician awareness of the appropriate therapy for cases of this potentially fatal Lyme carditis condition.

## CONCLUSION

Patients with significant new conduction abnormalities and suspected Lyme infection should be admitted for IV antibiotics and telemetry monitoring.^10^ This should be continued until either serology rules out Lyme, or in the case that Lyme is confirmed, until conduction abnormality improves to 1st degree AV block. The safe transition to oral antibiotics is indicated for the remainder of 21-28 days of antibiotic therapy. Considering the potentially fatal outcome of untreated Lyme disease, this approach is reasonable. In so doing, we as healthcare providers can work to minimize the unnecessary burden of a curable disease, avoid invasive procedures, and decrease the financial burdens of associated complications.

Primary care and hospital based clinicians generally need to initiate aggressive antibiotic therapy in patients presenting with possible Lyme carditis.^10^ Even when obvious or confirmed exposure to ticks is absent, it would behoove clinicians to treat patients aggressively until this disease is ruled out when symptoms are suggestive of Lyme disease. For this particular patient, it was fortunate that his misdiagnosed and mismanaged condition during both ED visits and initial hospital admission did not result in permanent comorbidity. This case report highlights a rare, albeit present, clinical knowledge gap which warrants special attention in view of the emerging public health concern of Lyme disease in Michigan.

### Conflict of Interest

The authors have no financial or other conflict to interest disclosures to make.
